# Colonization of *Clostridium butyricum* in Rats and Its Effect on Intestinal Microbial Composition

**DOI:** 10.3390/microorganisms9081573

**Published:** 2021-07-23

**Authors:** Xianshu Luo, Qing Kong, Yuming Wang, Xuefeng Duan, Peng Wang, Chenman Li, Yuchen Huan

**Affiliations:** School of Food Science and Engineering, Ocean University of China, Qingdao 266003, China; 17864260858@163.com (X.L.); wangyuming@ouc.edu.cn (Y.W.); oucdxf@163.com (X.D.); wangp960517@163.com (P.W.); dawnlcm@163.com (C.L.); huanyuchen79@outlook.com (Y.H.)

**Keywords:** *Clostridium butyricum*, real-time quantitative PCR, colonization, gut microorganisms

## Abstract

Gut microorganisms participate in many physiological processes. In particular, *Clostridium butyricum* can modulate gut microorganisms and treat diseases. The colonization and persistence of strains in the gut contribute to beneficial effects, and the colonization by *C. butyricum* in the gut is currently unknown. We investigated the total intestinal contents of *C. butyricum* at 12 h, 24 h, 48 h, and four and six days using real-time reverse transcription-PCR, after oral administration of *C. butyricum* to rats for seven consecutive days. We assessed the bacterial community structure using Illumina MiSeq sequencing. The results showed that *C. butyricum* was mainly colonized in the colon. The total content of *C. butyricum* in the gut increased significantly at 12 h after administration. Exogenous *C. butyricum* could still be detected in the gut six days after administration. Administration of *C. butyricum* significantly enhanced gut microbial diversity. The relative abundance of short-chain fatty acid-producing bacterial genera was shown to be higher than that of the control group, and treatment with *C. butyricum* elevated Firmicutes and diminished Bacteroidetes phyla compared with to the control group. These findings laid the foundation for the study of probiotic colonization capacity and the improvement of microflora for the prevention of gut diseases.

## 1. Introduction

The gut microbiome is an essential component of organisms, involved in many host physiological processes [1,2]. Indigenous bacteria in the mammalian gut have long been appreciated for their potential benefits to the host: provision of essential nutrients, metabolism of indigestible compounds, resistance to colonization by opportunistic pathogens, and contribution to the development of intestinal structure [3]. Healthy gut microbiota is essential for the overall health of humans and animals. The proper bacterial composition of the human microbiota prevents the development of disease, many metabolic diseases, inflammatory bowel diseases and even cardiovascular diseases have been reported to be associated with alterations in the microbial composition of the gut [4,5]. This homeostasis was achieved through interaction between the gut microbes and the host.

*Clostridium* is an anaerobic, Gram-positive, butyrate-producing bacillus. *C. butyricum* resides in the gastrointestinal tract and has a protective role against pathogenic bacteria and intestinal injury, via modulation of gut microbial metabolites [6], such as short-chain fatty acids (SCFAs) [7]. Duncan reported that *C. butyricum* could directly replenish butyrate as a butyrate-producing probiotic during repair of the damaged intestinal mucosa [8]. Obesity is associated with dysbiosis of intestinal flora, enhanced inflammation, poor barrier function, and reduced SCFA production, while butyrate-producing *C. butyricum* has been shown to treat the onset of high-fat diet-induced obesity and improve obesity-associated insulin resistance [9].

Probiotic-host interactions contribute to many physiological processes, including nutrient acquisition, development of the gut-specific immune system, and protection against infectious pathogens [2]. The World Health Organization defines probiotics as live microorganisms that, when administered in sufficient amounts, confer health benefits to hosts [10]. Successful colonization of the gut tract is a key factor in the ability of probiotics to exert sufficient host interactions to deliver health benefits. Although several studies have shown that the application of probiotics does not often lead to colonization, the survival of probiotics in the gut and their persistence in the host is a strong criterion for the successful effects of probiotics. The current literature shows that the probiotic bacteria *Bacillus sp.*, *Enterococcus sp.*, and *Pediococcus sp.* strain Ab1 can successfully colonize the digestive tract of different fish species, and *P. acidilactici* can remain in the intestine for at least 17 days without continual dietary administration [11]. However, some studies have also shown that probiotics ingested by humans are excreted in large quantities shortly after drug administration [12]. Similarly, Grimm et al. indicated that *Bifidobacteria* could not colonize the intestine of specific pathogen-free mice, but were able to colonize the intestine of germ-free mice [13]. An explanation for these results could be that competitive exclusion of species-native gut flora and individual differences among human subjects limit exogenous probiotic colonization. The colonization ability of different microorganisms varies, as does the colonization of the same microorganism in different individuals and sites of colonization. Many studies have reported beneficial effects of probiotic-host interactions on the host, and the native microorganisms in the host gut may have a long evolutionary history and have adaptive health properties to the host [14]. Therefore, it is reasonable to assume that local strains are better probiotic strains. As members of the autochthonous gut microbiota, it is likely that these species occupy specific niches that allow them to replicate and establish long-term stable populations [15].

*C. butyricum* is a native bacterium in the animal intestine, and the fate of *C. butyricum* in the intestine is a key bridge to study its interactions with the host. Studying the site of colonization and residence time of *C. butyricum* in the intestine is an essential part of exploring its effects on the host. There is a lack of research on the site of *C. butyricum* colonization and the effect of multiple gavage on the retention time of *C. butyricum* in the intestinal tract, except for Rumiko Sato [16], who studied the germination of *C. butyricum* in the intestine. Based on the above considerations, we established the first stable method (RT-qPCR) for detecting *C. butyricum* in the intestine and studied the colonization site of *C. butyricum* for the first time. Furthermore, we determined the retention time of exogenous *C. butyricum* in the intestine of specific pathogen-free Wistar rats for the first time and explored its effect on the microbial composition of the colon.

## 2. Materials and Methods

### 2.1. C. butyricum Cultivation

*C. butyricum* ZJUCB was preserved at the Ocean University of China. A thermostatic incubator (SHP-250, JINGHONG, Shanghai, China) was used to cultivate *C. butyricum* in Reinforced Clostridium Medium for 24 h at 37 °C. 16S rDNA sequencing (27F: AGAGTTTGATCCTGGCTCAG, 1492R: TACGGCTACCTTGTTACGACTT) was performed on the broth of *C. butyricum*, and the sequencing results were compared on the NCBI website (https://www.ncbi.nlm.nih.gov; accessed on 25 December 2020) to confirm that the strain cultivated was *C. butyricum*. The *C. butyricum* fermentation broth was centrifuged at 10,000× *g* rpm for 10 min and 20% trehalose was added to the bacterial pellets as a protective agent before freeze-drying. Freeze-dried bacteria were counted using the blood counting chamber, with a result of 10^10^ cfu/mL. The freeze-dried powder was stored until usage.

### 2.2. Primers and PCR Amplification

Primer sets developed in this study were either from references or were designed based on the highly conserved regions of the genome using the GenBank database (https://www.ncbi.nlm.nih.gov/genbank, accessed on 19 July 2020) and using the DNAMAN for Windows, version 10. The primers were synthesized commercially by Ruibiotech. All primers used are in Table 1.

Each PCR mixture (20 μL) contained 10 μL 2 × Phanta Max Buffer, dNTP Mix (10 mM each), 0.8 μL Forward primer, 0.8 μL Reverse primer, 1 μL of bacterial template DNA, 0.4 μL Phanta Max Super-Fidelity DNA Polymerase (Vazyme, Vazyme Biotech Co., Ltd., Nanjing, China), and 6.6 μL nuclease-free water. Determining the optimal annealing temperature for each primer set (Table 1) and testing the specificity of each primer set was done by using Applied biosystems 2720 Thermal Cycler (Applied Biosystems, Thermo Fisher, Waltham, MA, USA). The PCR program consisted of 1 cycle with a DNA Pre- denaturation step at 95 ℃ for 30 s and 35 cycles with a DNA denaturation step at 95 ℃ for 15 s; followed by an annealing step for 15 s and elongation step at 72 °C for 45 s. The PCR was completed with a final elongation step at 72 °C (5 min).

### 2.3. Real-Time qPCR

Real-time quantitative PCR was performed on Thermo Lifetech ABI QuantStudio 3 from Applied Biosystems (Applied Biosystems, Thermo Fisher, America). All amplification reactions were carried out in 96-well optical-grade PCR plates in triplicate (Applied Biosystems, Thermo Fisher, America), each with 20 μL, sealed with optical sealing tape (Ruibiotech, Qingdao, China). Amplification reactions were done with 10 μL SYBR^®^ Green Realtime PCR Master Mix (QKD-201T, TOYOBO, Osaka, Japan), 0.8 μL Forward primer, 0.8 μL Reverse primer, 6.4 μL nuclease-free, and 2 μL of template DNA or nuclease-free (Primer sets was the same as PCR amplification). Controls without templates were included to ensure that no unspecific breakdown or amplification occurred. The amplification was done with the following temperature profiles: one cycle of 90 ℃ for 60 s; 40 cycles of 95 ℃ for 15 s and Annealing temperature for 60 s. Finally, melting curve analysis for assessing amplicon specificity. Quantitation was done by using standard curves made from known concentrations of pure culture *C. butyricum* containing the respective amplicon for each set of primers (Figure S1: Supplementary Materials). Efficiency was calculated from a linear regression curve through the data points, using the following equation: E = −1 + 10^(−1/slope)^. Primer set Cbtu-a showed satisfactory efficiency values ranging between 90 and 110% with an R^2^ higher than 0.999 (Figure S1).

### 2.4. Animals and Experimental Groups

All animal procedures were performed by the guidelines of the ethical committee of experimental animal care at the College of Food Science and Engineering, Ocean University of China.

Male Wister rats weighing 280 to 300 g were purchased from Jinan Pengyue Experimental Animal Breeding Co., Ltd., Jinan, China. Thirty Wistar rats were randomly assigned to six groups according to their body weight (n = 5): a control group (sterilized normal saline (NaCl 0.8%) was administered to the rats), and five test groups treated *C. butyricum* (*C. butyricum* freeze-dried powder dissolved in sterilized normal saline (10^8^ CFU/mL) were administered to the rats). Test groups included: rats were sacrificed on day 6 (group A), on day 4 (group B), on day 2 (group C), on day 1 (group D), at 12 h (group E), after 7 consecutive days of gavage with *C. butyricum*. Rats were kept in individually ventilated cages in a pathogen-free animal facility at a temperature (20–22 °C), humidity (50–60%), and a pre-set light-dark cycle (12 h:12 h) was raised for 21 days. During the experimental periods, rats had free access to tap water and chow. Five animals were sacrificed for each observation at various time points (Table S1), and the entire intestinal tract, from the gullet to the rectum was removed. The contents of the cecum and the colon were completely flushed out with normal saline, and the contents were collected and stored at −80 °C until usage.

### 2.5. Genomic DNA Extraction from Pure Culture Strains and Fecal Samples of Rat Intestinal

Both pure cultured bacterial genomic DNA and lyophilized bacterial genomic DNA were extracted by the Tiangen bacterial genomic DNA extraction kit (DP302-02, TIANGEN, Beijing, China). Genomic DNA was extracted from fecal samples using the Tiangen fecal genomic DNA extraction kit (DP328-02, TIANGEN, Beijing, China). Fresh fecal samples were collected from the cecum and colon of each mouse under aseptic conditions, and they were immediately immersed in liquid nitrogen and stored at −80 °C until subsequent analysis. The genomic DNA from a fecal sample of the cecum and colon was extracted by the Tiangen fecal genomic DNA extraction kit (DP328-02, TIANGEN, Beijing, China) according to the manufacturer’s instructions. In all cases, the extracted DNA concentration was quantified by using a NanoDrop spectrophotometer ND 3.0 1000 (NanoDrop Technologies, Wilmington, DE, USA). The purity of the extracted DNA was checked by 1% agarose gel electrophoresis.

### 2.6. 16S rDNA V3-V4 Region Sequencing and Bioinformatics Analysis

The V3 and V4 regions of the 16S rRNA gene were sequenced on an Illumina Miseq platform at Majorbio Bio-Pharm Technology Co. Ltd. (Majorbio, Shanghai, China). On the Uparse platform (http://drive5.com/uparse/, accessed on 25 December 2020), the sequences obtained were picked into operational taxonomic units (OUT) by clustering 97% sequence similarity and classified at various taxonomic ranks (phylum, order, class, family, and genus) [18]. Alpha diversity and unweighted principal coordinate analysis plots using the phylogenic tree-based unifrac distance metric were generated using scripts from the QIIME package [19].

### 2.7. Statistical Analysis

Statistical analysis of the result of the six groups was performed using Students t-tests and a two-way ANOVA followed by Tukey’s post-hoc test was used to determine the significance. All RT-qPCR data and sequencing data were analyzed using GraphPad Prism Version 8.0 and the online Majorbio-Sanger cloud platform (https://cloud.majorbio.com, accessed on 25 December 2020).

The Illumina sequencing raw data were deposited in the NCBI sequence read archive database and assigned the accession no. PRJNA730420.

## 3. Results

### 3.1. Specificity of Primer

**Figure 1:**
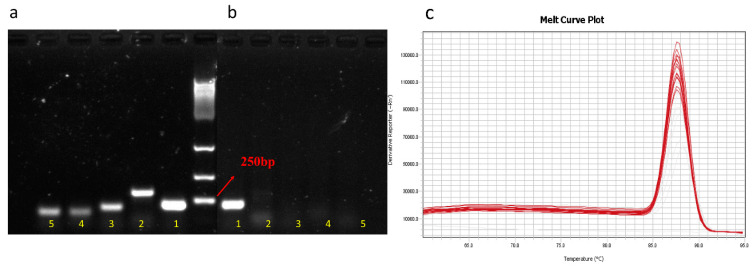
The primer set Cbtu-1 had high specificity. (**a**) All primer sets give positive results in a single system for PCR amplification (Primer sets 1, 2, 3, 4, and 5 are the same as Table 1). (**b**) Primer set Cbtu-1 amplifies the target product accurately in the complex system of intestinal microbial genomic DNA with a product length of 213 bp. (**c**) The melting curve plot of RT-qPCR amplification of primer set 1 is a single peak.

All primer sets used for PCR amplification of a single system of *C. butyricum* genomic DNA had high specificity, and only gave positive results for the corresponding target bacteria with the expected product size (Figure 1a). However, only one primer set, Cbtu-1, had high specificity in the complex PCR amplification system of intestinal microbial genomic DNA (Figure 1b,c).

### 3.2. Quantitation of C. Butyricum Populations in Rat Intestinal

**Figure 2:**
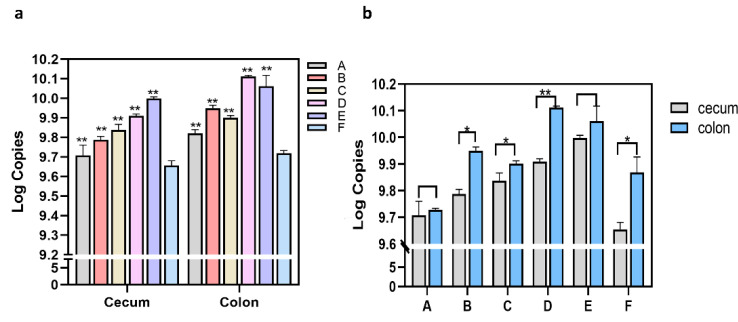
The level of *Clostridium butyricum* in the cecum and colon increased significantly after 7 consecutive days of gavage with *C. butyricum*. Raw data from RT-qPCR amplification were analyzed and plotted on GraphPad Prism Version 8.0 with two-way ANOVA to determine the significance. (**a**) Compared with group F, there is a highly significant increase in the colon, *C. butyricum* in the cecum have no significant increase on day 6 (group A) after administration. (**b**) *C. butyricum* is more abundant in the colon than in the cecum. Results are shown as mean ± SEM (n = 3). * *p* < 0.05 and ** *p* < 0.01 compared to the control group (group F). A: Day 6th after administration; B: day 4th after administration; C: day 2nd after administration; D: 24 h after administration; E: 12 h after administration; F: administration of sterile saline group (control).

RT-qPCR was used to detect changes in the quantity of *C. butyricum* in the cecum and colon at 12 h, 24 h, 48 h, and four and six days after oral administration of *C. butyricum* to rats for seven consecutive days and to determine colonization sites of endogenous *C. butyricum* in the intestine of specific pathogen-free Wistar rats. The results showed that the highest levels of *C. butyricum* were found in the cecum and colon at 12 h after seven consecutive days of *C. butyricum* administration. *C. butyricum* content was significantly increased in the cecum and colon at 24 h, 48 h, and day 4 after administration compared to the control group administered sterile saline for seven consecutive days (*p* < 0.01) (Figure 2a). *C. butyricum* levels were lowest in the cecum of rats at six days after cessation of administration and increased in this group, but not significantly (*p* > 0.05) compared to the control group (Figure 2a). It was found that the content of *C. butyricum* in the colon was highest after administration of both sterile saline and exogenous *C. butyricum* by comparing *C. butyricum* in the cecum and colon (Figure 2b). In summary, both the cecum and colon, were the sites of colonization of endogenous *C. butyricum*, but the content in the colon was higher than that in the cecum. Second, the content of *C. butyricum* in the intestine was maintained at a higher level for at least six days after continuous administration of *C. butyricum* to rats.

### 3.3. C. butyricum Changes the Composition of Rat Intestinal Flora

**Figure 3:**
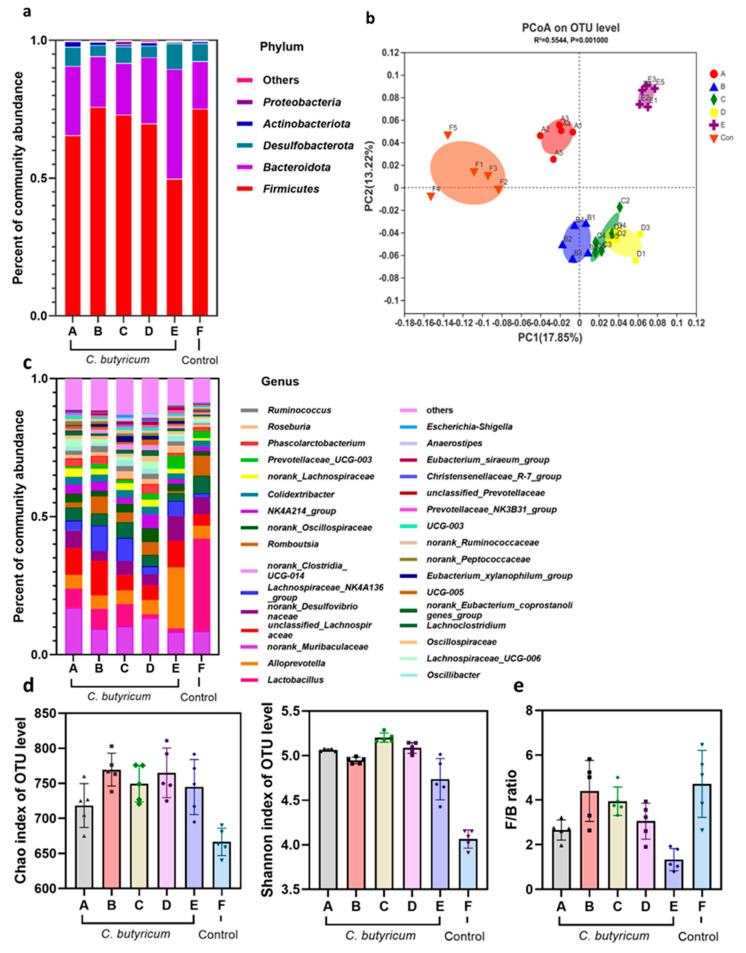
*C. butyricum* alters the intestinal flora of Wistar rats. Community abundance percentage plots showing the bacterial phyla and genera most differing in abundance between the treatment groups. (**a**,**c**) The colors of community abundance percentage plots represent the relative percentage of microbial families assigned to each sample. (**b**) Beta diversity of gut microbes analyzed by PCoA. PCoA used unweighted unifrac analysis to calculate the distances between the 6 groups in the colonic stool samples. The percentage shown on each axis explains the proportion of each dimension. (**d**) 16S rDNA V3-V4 sequencing to evaluate Chao richness and Shannon diversity. (**e**) The *Firmicutes*/*Bacteroidetes* ratio was calculated as a biomarker of intestinal flora dysbiosis. A: day 6th after administration; B: day 4th after administration; C: day 2nd after administration; D: 24 h after administration; E: 12 h after administration; F: administration of sterile saline group (control).

The composition of the rat intestinal flora was studied via sequencing the 16S bacterial genes. The unweighted UniFrac principal co-ordinate (PCoA) analysis showed that the composition of the intestinal flora of rats intragastrically administered with *C. butyricum* was different from that of the control group (Figure 3b). This was also evidenced by the differences between the experimental and control groups at the gate and genus levels (Figure 3a,c). By assessing the diversity of α, it was found that after administration of *C. butyricum* lyophilized powder for seven consecutive days in rats, there was a significant difference between the intestinal microbial richness and diversity (*p* < 0.01) (Figure 3d).

Firmicutes and Bacteroides were found to be the most abundant phyla in intestinal microbes (Figure 3a). When lyophilized *C. butyricum* powder was administered to rats for seven consecutive days, the ratio of Firmicutes and Bacteroides in the intestinal microbes of the experimental group changed significantly (*p* < 0.01). The changes in the ratio of Firmicutes/Bacteroides in group E (rat intestinal microbes at 12 h after gavage) were the largest, and Bacteroides increased by about two times compared to the control group (Group E: 40.47%, Con group: 17.26%) (Figure 3e). Interestingly, after observing the experimental data of groups A, B, C, and D, we found that after the administration of *C. butyricum*, with the extension of feeding time, the ratio of Firmicutes/Bacteroides was gradually recovered and, by day 6, the content of Bacteroides and Firmicutes in rats had returned to normal levels (Figure 3e).

**Figure 4:**
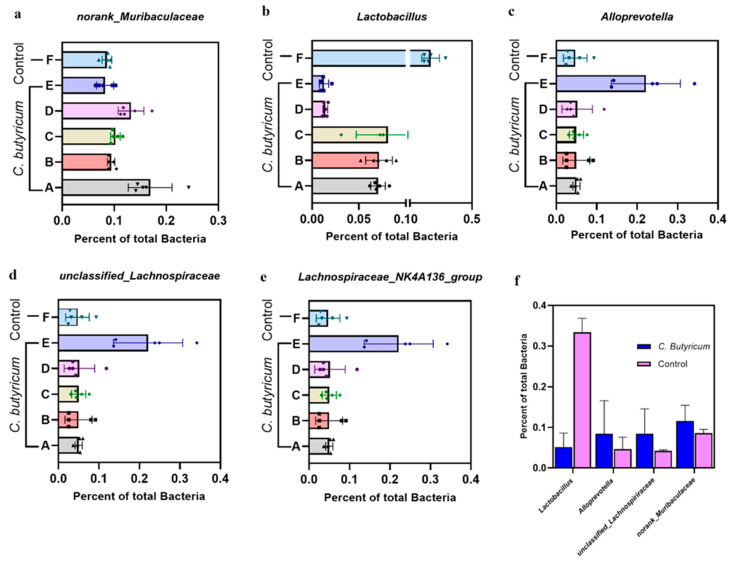
Histogram of the percentage of bacterial genera showing the differences in the abundance of the top five bacterial genera between treatment groups. (**a**–**e**) The genera *norank_Muribaculaceae*, *Lactobacillus*, *Alloprevotella*, *unclassified_Lachnospiraceae*, and *Lachnospiraceae_NK4A136_group* changed significantly after gavage of *C. butyricum*. (**f**) Changes in the relative abundance of the genera *norank_Muribaculaceae*, *Lactobacillus*, *Alloprevotella*, *unclassified_Lachnospiraceae*, and *Lachnospiraceae_NK4A136_group* in the administered group compared to the control group. A: day 6th after administration; B: day 4th after administration; C: day 2nd after administration; D: 24 h after administration; E: 12 h after administration; F: administration of sterile saline group (control).

We then evaluated the differences in the gut microbes of each group at the genus level in different periods and found that some genera showed significant changes (*p* < 0.01). First, the percentage of *Lactobacillus* in the intestinal microbes of the experimental group decreased by about six-fold compared to the control group (experimental group: 5.07%; control group: 33.35%) (Figure 4b,f). Conversely, the content of other bacteria producing butyrate, acetate, and propionate increased. For example, the percentage of *Alloprevotella* increased from 4.70% to 8.51% (Figure 4c), the percentage of *unclassified_Lachnospiriraceae* increased from 4.19% to 8.52% and the percentage of *norank_Muribaculaceae* increased from 8.58% to 11.65% (Figure 4a,d). Second, we analyzed the composition of intestinal microbes at different time points after administration of *C. butyricum* and found that the relative abundance of *unclassified_Lachnospiriraceae* and *Lachnospiriraceae_NK4A136_group* showed an increasing trend within four days after administration of *C. butyricum*. By day 6, the relative abundance began to decrease (Figure 4d,e). However, *norank_f muribaculaceae* continued to increase on day 6 after administration, and the relative abundance of *norank_f muribaculaceae* was approximately twice that of the control group (16.96% on the day 6) (Figure 4a). Notably, an increase in the relative abundance of *Allprevotella* only appeared 12 h after gavage. After 24 h, it returned to the normal level, and the relative abundance of *Allprevotella* remained at the normal level after 48 h and four and six days (Figure 4c). Overall, continuous gavage of *C. butyricum* changed the relative abundance of intestinal microbes in rats within a certain period, and the experimental results showed that some effects persisted.

## 4. Discussion

Intestinal flora is altered in many diseases [20]. For example, inflammatory bowel disease, hypertension, obesity, and diabetes are closely related to intestinal flora imbalance [21,22,23,24]. Probiotics are an effective way to regulate intestinal flora imbalance and can be used as an effective treatment for many diseases. *C. butyricum* exists in the intestine and has many beneficial functions [25,26,27]. This study aimed to provide some further evidence for the possibility that *C. butyricum* can be used as a probiotic, by clarifying the site of colonization, the persistence of exogenous *C. butyricum*, and its effect on microbial composition in the intestine.

We established a method to detect the content of *C. butyricum* in the intestinal tract, showing the relative abundance of *C. butyricum* in the cecum and colon after oral administration of *C. butyricum* to rats for seven consecutive days and the distribution of endogenous *C. butyricum* in the intestine. In addition, we have expanded the existing knowledge on the regulation of the richness and diversity of gut microbes. *C. butyricum* protects the intestinal flora of colon and rectal cancers [28]. Kanai et al. and Seo et al. have shown that the presence of *C. butyricum* in the intestine inhibits pathogenic bacteria and protects against intestinal injury [6,28]. To carry out their functional activities, *C. butyricum* must be able to survive in the gastrointestinal tract and persist, at least transiently, in the host. *Bacillus subtilis* spores can germinate and even transiently colonize the intestine. However, the spores themselves might also exert an immunostimulatory effect to exclude colonization of the intestine by harmful pathogens [29]. This suggested that using exogenous *C. butyricum* by administering it to specific pathogen-free Wistar rats to extend the probiotic potential of *C. butyricum* in this study was theoretically valid. A previous study by Rumiko Sato [16] showed that *C. butyricum* spores tolerated gastric acid but did not germinate in the front of the stomach and small intestine due to low pH and high oxidation-reduction potential. *C. butyricum* spores germinated in the proximal and middle small intestine, cecum, and colon; at the same time, the oxidation-reduction potential in the intestine was immediately reduced after the spores germinated [30]. The findings in this study showed that after administration of exogenous *C. butyricum* lyophilized powder for seven consecutive days, the content of *C. butyricum* in the cecum and colon of rats increased significantly (*p* < 0.01) compared to the control group. Administration of sterile saline in the control group showed that the colon was the colonization site of endogenous *C. butyricum*. Furthermore, although the content of *C. butyricum* in the cecum and colon decreased with increasing feeding time, there was still a significant increase in the content of *C. butyricum* in the colon compared to the control group at day 6 after administration (*p* < 0.01). Further, Yue et al. [31] studied *Lactobacillus casei* SY13 short-term oral administration (once in one day) and long-term treatment (once daily, 27 times) on *Lactobacillus casei SY13* colonization in the intestinal tract. The results showed that with short-term gavage, *Lactobacillus casei SY13* cells remained in the intestine for less than three days, which is consistent with the results of the current research. However, for long-term gavage, the cells were retained in the intestine for seven days. Long-term oral administration prolonged the retention time of this bacterium. In this study, to analyze the effect of multiple administrations of *C. butyricum* on the level of colonization in the gut, we administered it for seven consecutive days. We concluded that multiple administrations enhanced the ability of *C. butyricum* to colonize the intestinal tract. After multiple administrations of *C. butyricum* to rats, although some *C. butyricum* spores were excreted by intestinal motility before germination, some *C. butyricum* spores germinated and grew. A possible explanation for the difference in results was that when microorganisms were supplemented for a long time, they could colonize the intestine because their reproduction rate was higher than the expulsion rate. However, the possibility that the results of this study show temporal persistence could not be excluded. It was reported that *Bifidobacteria* could not stably colonize in the intestine of specific pathogen-free mice but were able to colonize the intestine of germ-free mice and the highest bacterial concentration in the colonic lumen [13]. The competitive exclusion of the intestinal native flora and the differences between individual subjects limit the colonization of exogenous probiotics, which could explain the phenomenon of time-dependent colonization of the intestine by exogenous probiotics. Conversely, many researchers believe that microorganisms of human origin are important criteria for the selection of probiotics [14,32]. Although most probiotic strains have been derived from human feces or intestines, they are gradually excreted from the body after administration is stopped or shortly afterward. Most *lactobacilli* found in the mammalian gut are not the true intestinal inhabitants; they are retained because they meet the nutrient requirements of the organism. The colon has the thickest mucus layer, and butyrate affects the composition of the mucus layer [33]. Intestinal bacteria have adapted to colonize the mucus layer by adhering to intestinal mucus components, using mucus-derived nutrients, and sensing chemical cues for adaptation [34]. Although no specific groups of mucus-adhering bacteria have been identified, there is evidence that bacteria can bind directly to mucins by expressing specific proteins, pili, fimbriae, and flagella [35]. Therefore, probiotics remain mainly in the colon. In conclusion, after supplementation with exogenous *C. butyricum* for seven consecutive days, the levels of *C. butyricum* in the cecum and colon were significantly higher than those in the control group due to a greater colonization rate than expulsion rate of *C. butyricum* in the intestine; *C. butyricum* was retained in the colon for at least six days.

Intestinal bacteria are a key factor in regulating the digestion in the gastrointestinal tract and have a key immune effect on the colonization of pathogenic bacteria. These microorganisms prevent bacterial invasion by maintaining the integrity of the intestinal epithelial cells [36]. The main phyla in the gut microbes are Firmicutes, Bacteroidetes, Actinobacteria, Proteobacteria, Fusobacteria, and Verrucomicrobia. Firmicutes and Bacteroides account for 90% of the intestinal flora [36,37]. To clarify the impact of *C. butyricum* colonization on the composition of intestinal microbes, we performed 16S rDNA gene sequencing. Bioinformatics analysis showed that the composition of gut microbes was altered by *C. butyricum*. *C. butyricum* elevated Firmicutes and diminished Bacteroidetes phyla, compared to the control group. *Lactobacillus* in the gut showed a significant decrease after administration compared to the control group, which explains why Firmicutes showed a significant decrease after *C. butyricum* supplementation. Here, we also observed that the ratio of Firmicutes/Bacteroidetes decreased at 12 h after administration, but at 24 h, the abundance of Firmicutes and Bacteroides gradually recovered. At day 6, the abundances of Firmicutes and Bacteroides returned to normal levels, but their proportions still showed an increase compared to the control group. The ratio of Firmicutes to Bacteroides is a sign of gut health. As previously shown, hypertension, obesity, and inflammatory bowel disease are associated with an increased ratio of Firmicutes/Bacteroides [25,26,28,38]. After administration, the intestinal flora gradually recovered because healthy rats had a complete composition of gut microbes, and undisturbed intestinal immune function played an important role. We also found that *C. butyricum* increased the abundance of SCFA-producing bacteria, such as *unclassified_f Lachnospiriraceae*, *Lachnospiriraceae_NK4A136_group*, *Alloprevotella*, and *norank_f muribaculaceae*. The composition of the intestinal microbiota differs at different times after administration. The relative abundance of colonic *Alloprevotella*, *Lachnospiraceae*_*NK4A136_group* and *unclassified_Lachnospiraceae* increased and reached its highest level at 12 h after administration. While *norank_muribaculaceae* continued to increase on day 6 after intragastric administration, their relative abundance was highest on day 6. Hou et al. reported that *norank_f muribaculaceae* and *Alloprevotella* are negatively associated with obesity [18,39]. Zhang et al. pointed out that the main metabolites of *Lachnospiriraceae* is butyrate [40]. The abundance of the genera *Alloprevotella* and *Lachnospiraceae_NK4A136_group* abundance has been shown to be negatively correlated with inflammation [41]. Some studies have also proposed that increased *Alloprevotella*, *Lachnospiraceae_NK4A136_group* abundance, and fecal SCFA content are linked to a decrease in TLR4 signaling and LPS levels [42,43]. SCFAs, including acetate, propionate, butyrate, isobutyrate, and valerate, are important metabolites and information molecules in the intestinal flora. They are produced within the intestinal lumen by bacterial fermentation of mainly undigested dietary carbohydrates. In the cecum and large intestine, 95% of the produced SCFAs are rapidly absorbed by the colonocytes, while the remaining 5% are secreted in the feces [44,45]. SCFAs are the main energy source for epithelial cells and have many beneficial effects, such as improving barrier function and reducing mucosal inflammation [46]. In general, we believe that *C. butyricum* can play a beneficial role through its metabolites (mainly SCFAs) or by increasing the abundance of other SCFA-producing bacteria. After supplementation with exogenous *C. butyricum*, different genera of SCFA-producing bacteria in the intestine were increased at different times, with some showing short-term changes rapidly increasing after administration, while others increased in abundance some time after administration. Despite the differences in the time of appearance, the effects on intestinal microorganisms were definite. This result suggested that supplementation with exogenous *C. butyricum* promoted an increase in the relative abundance of SCFA-producing bacteria in the intestine, prolonging their beneficial effects on the host.

## 5. Conclusions

Exogenous bacteria have difficulties colonizing the intestine because of competitive exclusion and individual differences. *C. butyricum*, a native intestinal bacterium, has the advantage of occupying specific niches in the intestine, but colonization is still difficult. In this study, we attempted to increase the level of *C. butyricum* colonization in the intestine and prolong the retention time of exogenous probiotics in the intestine by regulating the proliferation and expulsion rates. These results were reported by us: (1) *C. butyricum* is a native microorganism in the intestines and mainly colonizes the colon; (2) after gavage of *C. butyricum* to rats for seven consecutive days, it was retained in the intestinal tract for at least six days; and (3) *C. butyricum* changed the composition of the gut microbes.

This research suggested that *C. butyricum* is an intestinal native bacterium and that long-term intragastric administration facilitates colonization of *C. butyricum* in the colon. After seven consecutive days of exogenous *C. butyricum* supplementation, temporal colonization levels were observed during and at least six days after dietary supplementation. The residence of exogenous *C. butyricum* in the intestine altered the F/B ratio, a marker of intestinal health. In addition, the abundance of SCFAs (acetate, butyrate, and propionate)-producing bacteria in the colon increased. However, a significant decrease in the abundance of *Lactobacillus* was observed. A shortcoming of our study was that after seven consecutive days of feeding exogenous *C. butyricum*, the feeding time was only six days. Based on this, we could not conclude with absolute certainty that *C. butyricum* colonized the colon. However, we believe that continuous supplementation with exogenous *C. butyricum* could prolong its residence time in the intestine. The presence of probiotics in the intestine might stimulate the beneficial effect of probiotics, but it has not been reported in the literature that probiotics need to colonize to exert health benefits on the host. This study prolonged the retention time of *C. butyricum* in the gut by adjusting the ratio of multiplication rate to expulsion rate and temporal colonization of *C. butyricum* in the gut. This would be a breakthrough point for the improvement of the health benefits of *C. butyricum*. As suggested in the study, the persistence of exogenous *C. butyricum* in the gut and the alteration in healthy gut microbes may be an entry point to study the role of *C. butyricum* in the prevention of certain diseases. Future studies should explore the impact of *C. butyricum* on the control of metabolic diseases associated with SCFAs.

## Figures and Tables

**Table 1:** Primer sets used to detect *C. butyricum* in rat intestine.

	Primer Set	Primers Sequences (5′–3′)	Annealing Temp (°C)	Approximate Amplicon Size (bp)	Reference or Source
1	Cbtu-1F	GTGCCGCCGCTAACGCATTAAGTAT	72	213	[17]
	Cbtu-1R	ACCATGCACCACCTGTCTTCCTGCC			
2	Cbtu-2F	GCATCCAGCAGACTTAGCAG	63	275	This study
	Cbtu-2R	CTTCCGACTGTCTCATCTTC			
3	Cbtu-3F	CAATGGTTGTGAATGCTGAGG	60	142	This study
	Cbtu-3R	CCAACTATGCTCATTTCGCCC			
4	Cbtu-4F	CCTCAAATCCGCCTTCTGG	58	102	This study
	Cbtu-4R	GGATCTTGTTATCGTTCCG			
5	Cbtu-5F	GAGTCTGATTCGGTTGTGGC	60	135	This study
	Cbtu-5R	CCTCTCTTCCTTGAACTGGATG			

## Data Availability

The data that support the figures within this paper and other findings of this study are available from the corresponding author upon reasonable request.

## Supplementary Materials

The following are available online at https://www.mdpi.com/article/10.3390/microorganisms9081573/s1, Table S1: Administration time, execution time, and gavage dose for each group of rats. Figure S1: Amplification results of RT-qPCR, including amplification plot and melt curve plot of the standard curve.
