# Supplementary material for: Colonization of Clostridium butyricum in Rats and Its Effect on Intestinal Microbial Composition
Source: Microorganisms. 2021 Jul 23;9(8):1573. doi: 10.3390/microorganisms9081573 (PMC8401576; doi:10.3390/microorganisms9081573)
Supplement: Supplementary file 1 [file microorganisms-09-01573-s001.zip › microorganisms-1292965-supplementary.pdf]

microorganisms

Colonization of *Clostridium butyricum* in rats and its effect on intestinal microbial composition

Xianshu Luo, Qing Kong, Yuming Wang, Xuefeng Duan, Peng Wang, Chenman Li, Yuchen Huan

College of Food Science and Engineering, Ocean University of China, Qingdao 266003, Shandong, China

***Correspondence:** Dr. Qing Kong, School of Food Science and Engineering, Ocean University of China, Qingdao. Yushan Road 5, 62 Building, Qingdao, Shandong 266003, China

E-mail: kongqing@ouc.edu.cn

Tel: +86-532-8203-2290

Fax: +86-532-8203-2389

Table S1. Gavage schedule (The concentration of *C. butyricum* solution was 10^8 cfu/ml; and 3×10^8 CFU *C. butyricum* by gavage in rats daily)

Group	Gavage volume (ml/Day)	Gavage time (Days)	Time of sacrificing (Days) (stopping gavage to sacrifice)
A	3	7	6
B	3	7	4
C	3	7	2
D	3	7	1
E	3	7	0.5
F(Control)	3	7	0

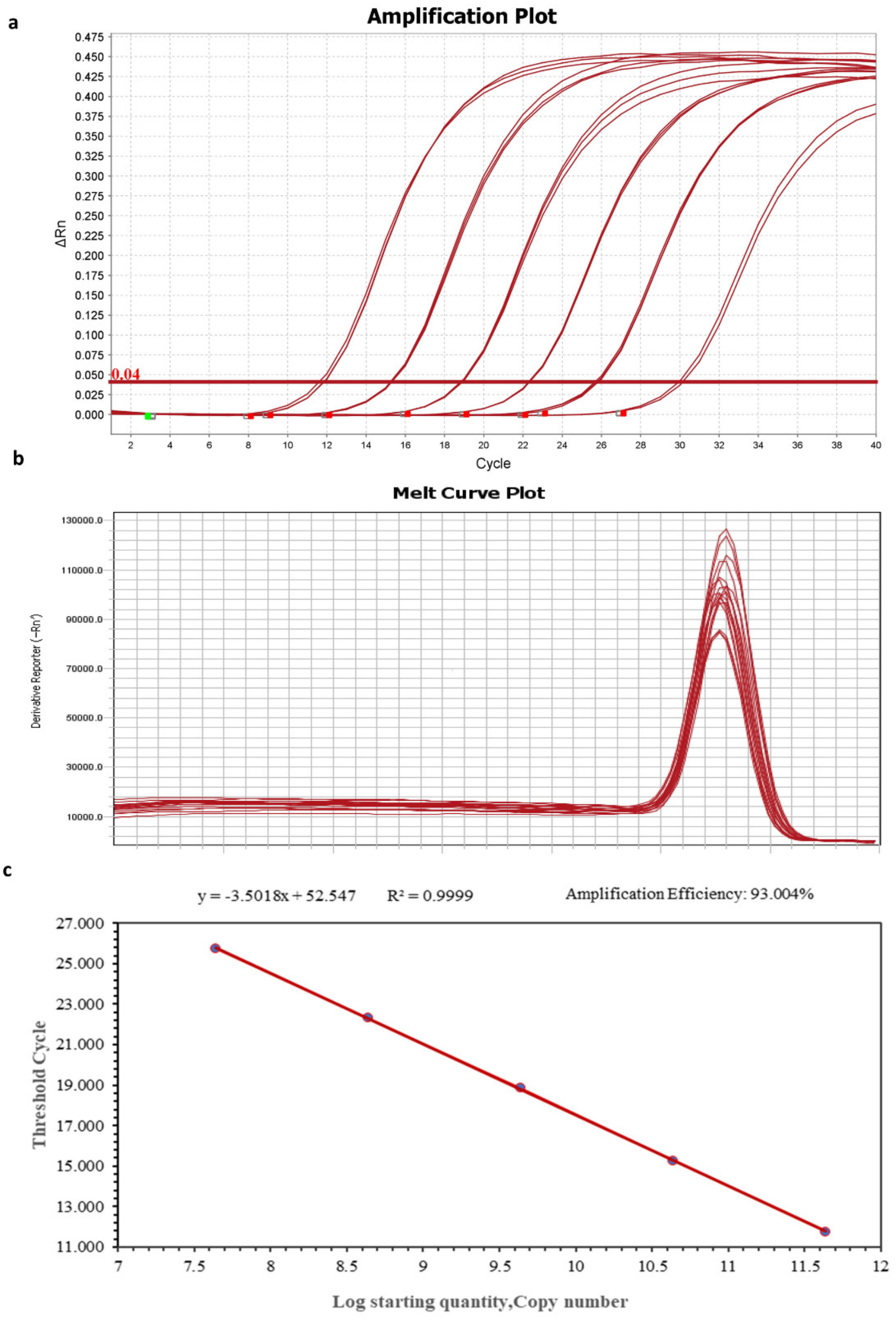

Figure S1. Preparation of standard curve. (**A**) PCR amplification curve of pure cultured *C. butyricum* genomic DNA after dilution using tenfold dilution method. (**B**) Melt curve polt of the standard curve.(**C**) Copy number logarithm of *C. butyricum* genomic DNA with the number of threshold cycles to establish the standard curve, amplification efficiency: 93.004%.
